# Standing Up for Inclusion of LGBTQIA+ Older Adults in Alzheimer’s and Parkinson’s Disease Research

**DOI:** 10.1093/geroni/igaf122.1095

**Published:** 2025-12-31

**Authors:** Jace Flatt

**Affiliations:** University of Nevada Las Vegas, Nevada, United States

## Abstract

Over 3 million adults aged 60+ in the U.S. identify as lesbian, gay, bisexual, transgender, queer, intersex, or another identity (LGBTQIA+). Yet, despite the growing population of LGBTQIA+ older adults, there are numerous unmet health and research challenges that, if addressed, would greatly benefit this community and those who support them. We will examine the unique risks and challenges LGBTQIA+ older adults face related to neurodegenerative disorders, including Alzheimer’s disease and related dementias and Parkinson’s disease. Drawing on lessons learned from our national research studies with LGBTQIA+ older adults, we will discuss key insights and critical gaps in knowledge and propose steps to ensure future research efforts and how to support emerging scholars in this field. This includes describing the current challenges in continuing this important research given today’s sociopolitical climate. We will also highlight the importance of collecting sexual orientation and gender identity (SOGI) data to ensure LGBTQIA+ aging communities are represented. In light of today’s sociopolitical climate, it’s more important than ever to advocate for inclusion of and to stand up for LGBTQIA+ aging communities and advocate for this critical research.

